# The association between hepatic viral infections and cancers: a cross-sectional study in the Taiwan adult population

**DOI:** 10.1007/s10238-023-01292-x

**Published:** 2024-01-27

**Authors:** Moreen Maliko, Fu-Hsiung Su, Abram Bunya Kamiza, Ming-Jang Su, Chih-Ching Yeh

**Affiliations:** 1 School of Public Health, College of Public Health, Taipei Medical University, 10F Biomedical Technology Building, No.301, Yuantong Rd., Zhonghe Dist., New Taipei City, 235603 Taiwan; 2 Department of Family Medicine, Cardinal Tien Hospital, Fu Jen Catholic University, New Taipei City, Taiwan; 3 School of Medicine, College of Medicine, Fu Jen Catholic University, New Taipei City, Taiwan; 4 Faculty of Health Sciences, Sydney Brenner Institute for Molecular Bioscience, University of the Witwatersrand, Johannesburg, South Africa; 5 The African Computational Genomic (TACG) Research Group, MRC/UVRI and LSHTM, Entebbe, Uganda; 6 Department of Clinical Pathology, Shuang Ho Hospital, Taipei Medical University, New Taipei City, 235041 Taiwan; 7 Department of Public Health, College of Public Health, China Medical University, Taichung, 406 Taiwan; 8 Cancer Center, Wan Fang Hospital, Taipei Medical University, New Taipei City, 116 Taiwan; 9 Master Program in Applied Epidemiology, College of Public Health, Taipei Medical University, New Taipei City, 235603 Taiwan

**Keywords:** Hepatitis B virus, Hepatitis C virus, Cancer, Adults, Cross-sectional study

## Abstract

**Background:**

Hepatitis B (HBV) and hepatitis C (HCV) viruses are diseases of global public health concern and are associated with liver cancer. Recent studies have revealed associations between hepatic viral infections and extrahepatic cancers. This study aimed to explore the associations between hepatitis B and C viruses and cancer at baseline in the Taiwan Biobank database while controlling for a wide range of confounding variables.

**Methods:**

In a cross-sectional study of adults aged > 20 years, we compared the distribution of demographic factors, lifestyle, and comorbidities between viral and nonviral hepatic groups using the chi-square test. Univariate and multivariate logistic regressions were performed to observe the associations between hepatitis B and C viral infections and cancers by estimating the odds ratio (OR) and 95% confidence interval (CI). Multivariate regression analysis was adjusted for sociodemographic factors, lifestyle, and comorbidities.

**Results:**

From the database, 2955 participants were identified as having HCV infection, 15,305 as having HBV infection, and 140,108 as the nonviral group. HBV infection was associated with an increased likelihood of liver cancer (adjusted OR (aOR) = 6.60, 95% CI = 3.21–13.57, *P* < 0.001) and ovarian cancer (aOR = 4.63, 95% CI = 1.98–10.83, *P* = 0.001). HCV infection was observed to increase the likelihood of liver cancer (aOR = 4.90, 95% CI = 1.37–17.53, *P* = 0.015), ovarian cancer (aOR = 8.50, 95% CI = 1.78–40.69, *P* = 0.007), and kidney cancer (aOR = 12.89, 95% CI = 2.41–69.01, *P* = 0.003).

**Conclusion:**

Our findings suggest that hepatic viral infections are associated with intra- and extrahepatic cancers. However, being cross-sectional, causal inferences cannot be made. A recall-by-genotype study is recommended to further investigate the causality of these associations.

## Introduction

Viral hepatitis infections are a global public health concern, with hepatic viruses B and C leading to chronic illness in millions around the world. Globally, it is estimated that 354 million individuals are infected with hepatitis B virus (HBV) or hepatitis C virus (HCV) [1]. In particular, the World Health Organization (WHO) estimates that 296 million people worldwide were living with chronic HBV infection, contributing to approximately 820,000 deaths in 2019 [2]. On the other hand, it was estimated that approximately 58 million individuals worldwide were living with chronic HCV infection in 2019. Additionally, 290,000 deaths attributed to HCV infection were reported [3]. The prevalence of HBV and HCV in the Taiwanese population has greatly declined from the elevated rates observed in the 1980s. This is primarily attributed to the introduction of the HBV mass vaccination campaign in 1984 and the extensive coverage of treatment.

In Taiwan, the prevalence of HBV is estimated to be approximately 9.7% in the adult population born before the implementation of the vaccination program [4]. Before this campaign, the prevalence of HBV was projected to be between 15 and 20% in the general population [5], with the major routes of spread being vertical and horizontal transmission in early life [6]. Among children, the HBV prevalence decreased from 10.5% before 1984 to 0.8% in 2007 [7]. Approximately 2–5% of the population was estimated to be infected with HCV [8–10], with a higher prevalence rate exhibited in the early 1980s, mainly attributed to the use of intravenous injections that were not properly sterilized [8]. HBV and HCV infections are the most common contributors to liver diseases, such as cirrhosis, liver cancer, and viral hepatitis-related deaths [1].

The current literature reveals that HBV and HCV are not only related to the development of liver cancer but extrahepatic cancers as well. Such associations have been observed in different cancer types, such as colorectal cancer [11–13], gallbladder and extrahepatic bile duct cancer [12, 14], pancreatic cancer [12–14], renal cancer [12], ovarian cancer [12], stomach cancer [13], oral cancer [13], anus cancer [14], skin cancer [14], and non-Hodgkin’s lymphoma [12–14]. Some inverse associations have been reported in uterine and prostate cancers [14]. The reported findings vary among studies and are therefore not entirely conclusive [11–14]. A number of studies were limited by insufficient control of environmental and behavioral confounders, small sample sizes, inadequate control of cancer-specific risk factors, and misclassification bias arising from the use of diagnostic tests with low sensitivity. This presents a need to explore these associations in large databases with sufficient control for sociodemographic, lifestyle, behavioral, and cancer-specific confounders.

Cancer is the leading cause of death in the Taiwanese population [15]. Therefore, it is imperative to explore the associations between hepatic viral infections and cancer to gain a deeper understanding of the associations, patterns, and risk factors relevant to the Taiwanese population. The Taiwan Biobank (TWB) presents a great opportunity to explore the associations between hepatic viral infections and cancers due to its large sample size, wide range of cancer-specific risk factors, and comprehensive ascertainment of HBV and HCV infections. Therefore, the main objective of this study was to explore the association between hepatic viral infections (HBV and HCV) and cancer at baseline using the TWB.

## Materials and methods

### Study population and design

**Fig. 1:**
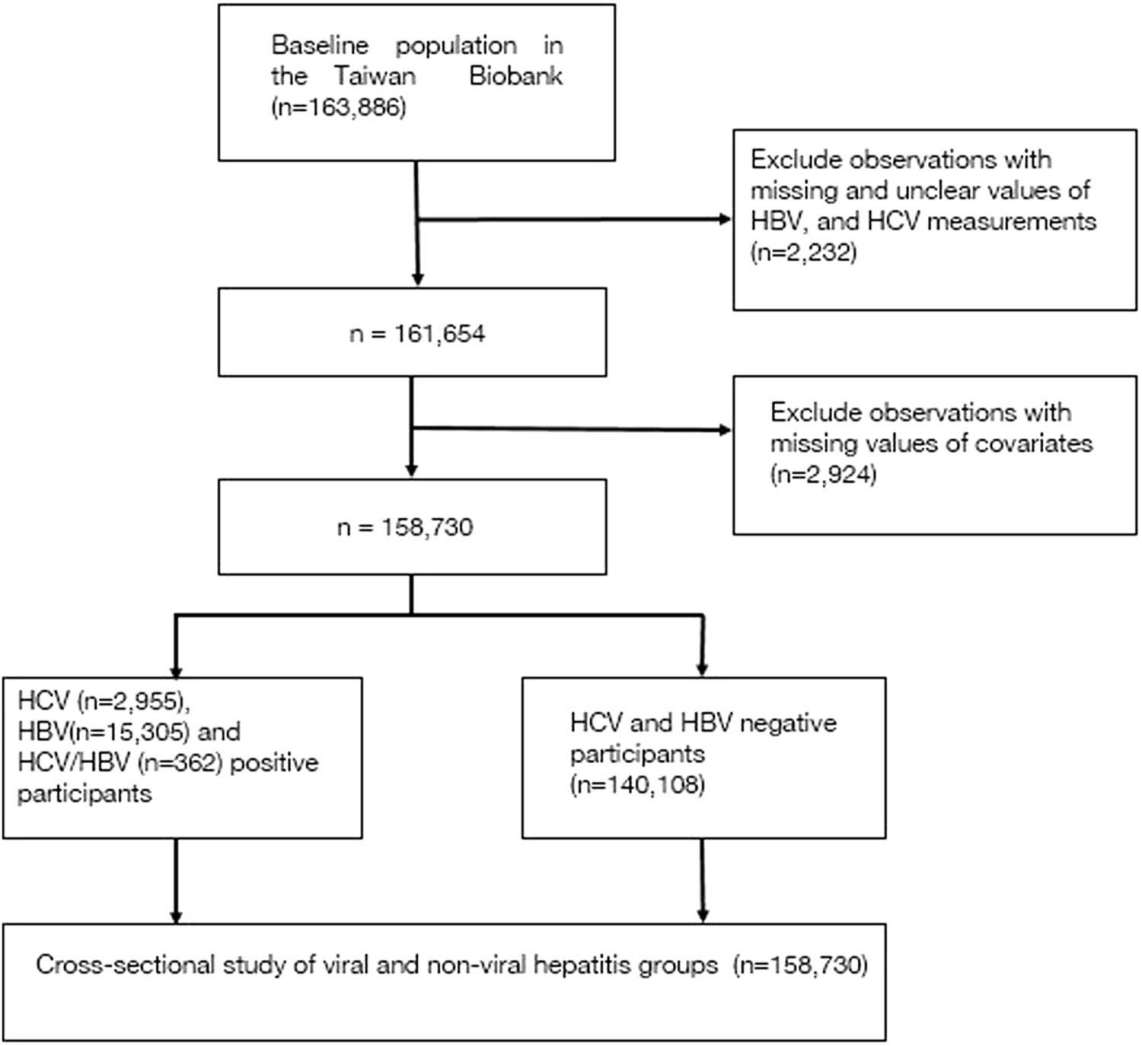
The study flow chart. Demonstrating the inclusion and exclusion criteria and determination of the final sample size

The TWB is an ongoing prospective study comprising adults aged 20 to 70 years at the time of enrollment. It currently has over 160,000 participants with a target of enrolling up to 200,000 participants recruited from different regions all over Taiwan. The database contains comprehensive information on the phenotypes of the consenting participants obtained at the baseline and follow-up visits through a structured questionnaire and physical and biomarker measurements [16]. This study employed a cross-sectional design to explore the baseline associations between different cancer types and hepatic viral infections with data collected from 2012 to 2022. A total of 163,886 participants were included in the study. We excluded participants with missing or unclear information on HBV and HCV measurements (n = 2232) and the required covariates (n = 2924). The final sample size was 158,370 participants. Of these, 2955 had HCV alone, 15,305 had HBV alone, 362 had HCV/HBV dual infection, and the nonviral hepatitis group comprised 140,108 participants (Fig. 1). The data provided were deidentified.

### Data

The database contains sociodemographic variables, such as age, sex, marital status, highest education level, residential area, income status, maternal and paternal origin, and anthropometric measurements, such as body weight and height. Sex was considered a binary variable (male and female) defined by birth characteristics. Age (in years) was categorized as < 50, 50–59, and ≥ 60 years. An individual’s marital status was considered single, married, or separated. The highest education levels were ‘junior high school and below’, ‘senior high school’, and ‘college and above’. Participant residences were classified as either ‘urban’ or ‘rural,’ based on their residential degree of urbanization. Maternal and paternal origins were grouped into ‘Hakka’, ‘Hokkien’, and ‘others’. Body mass index (BMI) was computed and categorized into low (< 18.5 kg/m^2^), normal (18.5–24.0 kg/m^2^), and ‘overweight and above’ (> 24.0 kg/m^2^). Income status was classified into three categories: < 30,000, 30,000–60,000, and ≥ 60,000 National Taiwan dollars (NTD) [17]. The recruitment period was divided into 3 categories i.e. 2012–2015, 2016–2019 and 2019–2022. Alcohol consumption status was categorized as ‘No’ for never drinkers and ‘Yes’ for previous and current drinkers. Smoking experience was regarded as ‘No’ for never smokers and ‘Yes’ for previous and current smokers. Exercise regularity was considered ‘No’ for participants without the habit of regular exercise and ‘Yes’ for those who had incorporated regular exercise. Betel nut experience was considered ‘No’ for those who reported never chewing betel nuts and ‘Yes’ for those who had previous or current experience. Information on comorbidities, such as hypertension, hyperlipidemia, and diabetes, was obtained from the survey. A family history of cancer was considered either ‘yes’ or ‘no’ based on the information provided from the survey.

### Ascertainment of hepatic viral infections and cancers

Ascertainment of HBV status was performed through an immunological test of hepatitis B surface antigen (HBsAg) using electrochemiluminescence immunoassay (ECLIA) (Roche Diagnostics GmbH, Mannheim, Germany), a highly sensitive test. ‘Positive’ or ‘reactive’ results were considered HBsAg positive, and ‘negative’ or ‘nonreactive’ results were considered HBsAg negative. HCV serostatus was ascertained using the HCV antibody test, which was also performed using the ECLIA test. Cancer ascertainment was obtained from the information collected in the survey questionnaire. If a participant reported more than one cancer, the primary cancer was considered the first cancer type. Cancers whose frequency was below 10 were grouped together in the category ‘Other’.

### Statistical analysis

This study examined the association between cancer and hepatic viral infections (HBV and HCV) at the baseline visit using the TWB database. The comparison of demographic factors, lifestyle, and comorbidities between the viral and nonviral hepatic groups was performed using the chi-square test. Hepatitis infection status was categorized into no infection, HBV infection and HCV infection. Logistic regression analysis was performed at both the univariate and multivariate levels by estimating the odds ratio (OR) and 95% confidence interval (CI). Adjustments were made for covariates that showed significant differences in distribution at the multivariate level. In the initial analysis of the HBV group, comparisons were made between the nonviral and HBV-infected groups (including both HBV alone and HBV-HCV coinfection). Similar considerations were made in the analysis of the HCV group. A sensitivity analysis was carried out in which the dual infection group was dropped and hepatitis viral groups were treated as HBV alone and HCV alone. All statistical analyses were performed using SAS software (version 9.4; SAS Institute).

## Results

### Demographic characteristics

A total of 158,370 participants took part in the study. Of these, 15,305 (9.7%) had HBV infection alone, 2955 (1.9%) had HCV infection alone, 362 (0.23%) had a dual infection of both HBV and HCV, and 140,108 (88.4%) were in the nonviral group. From the primary comparison of characteristics between the HBV-infected and control groups, significant differences were observed in the following variables: age group, sex, education level, marital status, maternal and paternal origin, residence, BMI, income level, family history of cancer, alcohol drinking, smoking experience, betel nut experience, exercise regularity, recruitment period, hyperlipidemia, and diabetes (Table 1). Compared with the nonviral hepatitis group, participants in the HBV infection group were generally more likely to be female, of Hokkien maternal and paternal origin, urban residents, within the BMI category ‘overweight and obese’, with a family history of cancer, smoking experience, and diabetes. Participants with HBV infection were also more likely to be in the age group 50–59 years, with a maximum education level of senior high school, and with income status > 60,000 NTD (Table 1).

**Table 1:** Distribution of socio-demographic, behavioral factors, and comorbidities between the HBV and nonviral groups

Variables	No HBV		HBV infected		*P*^c^
	(N = 140,108)		(N = 15,667)		
	N	(%)^a^	N	(%)^b^	
Sex					< 0.0001
Male	50,186	(35.82)	6524	(41.64)	
Female	89,922	(64.18)	9143	(58.36)	
Age group					< 0.0001
< 50	411	(0.29)	3	(0.02)	
50–59	72,857	(52.00)	8067	(51.49)	
≥ 60	66,840	(47.71)	7597	(48.49)	
Education level					< 0.0001
Junior high school and below	15,507	(11.07)	1941	(12.39)	
Senior high school	38,342	(27.37)	4744	(30.28)	
College	86,259	(61.57)	8982	(57.33)	
Marital status					< 0.0001
Single	25,130	(17.94)	1977	(12.27)	
Married	97,528	(69.61)	11,767	(75.11)	
Separated	17,450	(12.45)	1912	(12.62)	
Maternal origin					< 0.0001
Hokkien	106,720	(76.17)	12,715	(81.16)	
Hakka	21,458	(15.32)	2231	(14.24)	
Others	11,930	(8.51)	721	(4.60)	
Paternal origin					< 0.0001
Hokkien	98,244	(70.12)	11,741	(74.94)	
Hakka	20,071	(14.33)	2036	(13.00)	
Others	21,793	(15.55)	1890	(12.06)	
Residence					< 0.0001
Rural	45,065	(32.16)	4719	(30.12)	
Urban	95,043	(67.84)	10,948	(69.88)	
BMI					0.0043
Low	4999	(3.57)	495	(3.16)	
Normal	68,616	(48.97)	7576	(48.36)	
Overweight and above	66,493	(47.46)	7596	(48.48)	
Income (NTD)					< 0.0001
< 30,000	83,927	(59.90)	9656	(61.63)	
30,000–60,000	37,401	(26.69)	3786	(24.17)	
≥ 60,000	18,780	(13.40)	2225	(14.20)	
Alcohol drinking					0.0038
No	127,981	(91.34)	14,203	(90.66)	
Yes	12,127	(8.66)	1464	(9.34)	
Family history of cancer					< 0.0001
No	90,053	(64.27)	9258	(59.09)	
Yes	50,055	(35.73)	6409	(40.91)	
Smoking experience					< 0.0001
No	113,291	(80.86)	12,348	(78.82)	
Yes	26,817	(19.14)	3319	(21.18)	
Betel nut experience					< 0.0001
No	132,435	(94.52)	14,591	(93.13)	
Yes	7673	(5.48)	1076	(6.87)	
Exercise regularity					0.0608
No	85,587	(61.09)	9691	(61.86)	
Yes	54,521	(38.91)	5976	(38.14)	
Hypertension					0.9195
No	60,055	(42.86)	6722	(42.91)	
Yes	80,053	(57.14)	8945	(57.09)	
Hyperlipidemia					< 0.0001
No	115,400	(82.37)	13,246	(84.55)	
Yes	24,708	(17.63)	2421	(15.45)	
Diabetes					0.0327
No	88,170	(62.93)	9723	(62.06)	
Yes	51,938	(37.07)	5944	(37.94)	
Recruitment period					< 0.0001
2012–2015	45,307	(32.34)	5833	(37.23)	
2016–2019	57,543	(41.07)	6444	(41.13)	
2020–2022	37,258	(26.59)	3390	(21.64)	

Abbreviations: HBV, Hepatitis B Virus; BMI, Body Mass Index; NTD, National Taiwan Dollar

^a^: Percentages among the none HBV and/or HCV infected participants

^b^: Percentages among the HBV infected participants

^c^: Comparing the Non-viral and HBV infected group

Comparing the HCV-infected group and the control group, significant differences were observed for the variables age group, education level, marital status, maternal and paternal origin, residence, BMI, income level, family history of cancer, alcohol drinking, smoking experience, betel nut experience, exercise regularity, recruitment period, hypertension, and diabetes (Table 2). Participants who were infected with HCV were most likely to be above 50 years old, having completed at least senior high school, married, of Hokkien origin, residing in urban areas, earning less than 30,000 NTD, and having a family history of cancer (Table 2).

**Table 2:** Distribution of socio-demographic, behavioral factors, and comorbidities between the HCV and nonviral groups

Variables	No HCV		HCV-infected		*P*^c^
	(N = 140,108)		(N = 3317)		
	N	(%)^a^	N	(%)^b^	
Sex					0.1754
Male	50,186	(35.82)	1226	(36.96)	
Female	89,922	(64.18)	2091	(63.04)	
Age group					< 0.0001
< 50	411	(0.29)	0	(0.00)	
50–59	72,857	(52.00)	1029	(31.02)	
≥ 60	66,840	(47.71)	2288	(68.98)	
Education level					< 0.0001
Junior high school and below	15,507	(11.07)	933	(28.13)	
Senior high school	38,342	(27.37)	1120	(33.77)	
College	86,259	(61.57)	1264	(38.11)	
Marital status					< 0.0001
Single	25,130	(17.94)	292	(8.80)	
Married	97,528	(69.61)	2353	(70.94)	
Separated	17,450	(12.45)	672	(20.26)	
Maternal origin					< 0.0001
Hokkien	106,720	(76.17)	2773	(83.60)	
Hakka	21,458	(15.32)	359	(10.82)	
Others	11,930	(8.51)	185	(5.58)	
Paternal origin					< 0.0001
Hokkien	98,244	(70.12)	2559	(77.15)	
Hakka	20,071	(14.33)	375	(11.31)	
Others	21,793	(15.55)	383	(11.55)	
Residence					< 0.0001
Rural	45,065	(32.16)	751	(22.64)	
Urban	95,043	(67.84)	2566	(77.36)	
BMI					< 0.0001
Low	4999	(3.57)	98	(2.95)	
Normal	68,616	(48.97)	1515	(45.67)	
Overweight and above	66,493	(47.46)	1704	(51.37)	
Income (NTD)					< 0.0001
< 30,000	83,927	(59.90)	2372	(71.51)	
30,000–60,000	37,401	(26.69)	610	(18.39)	
≥ 60,000	18,780	(13.40)	335	(10.10)	
Alcohol drinking					< 0.0001
No	127,981	(91.34)	2876	(86.70)	
Yes	12,127	(8.66)	441	(13.30)	
Family history of cancer					< 0.0001
No	90,053	(64.27)	1904	(57.40)	
Yes	50,055	(35.73)	1413	(42.60)	
Smoking experience					< 0.0001
No	113,291	(80.86)	2439	(73.53)	
Yes	26,817	(19.14)	878	(26.47)	
Betel nut experience					< 0.0001
No	132,435	(94.52)	2966	(89.42)	
Yes	7673	(5.48)	351	(10.58)	
Exercise regularity					< 0.0001
No	85,587	(61.09)	1823	(54.96)	
Yes	54,521	(38.91)	1494	(45.04)	
Hypertension					0.0350
No	60,055	(42.86)	1361	(41.03)	
Yes	80,053	(57.14)	1956	(58.97)	
Hyperlipidemia					0.0793
No	115,400	(82.37)	2771	(83.54)	
Yes	24,708	(17.63)	546	(16.46)	
Diabetes					< 0.0001
No	88,170	(62.93)	1929	(58.15)	
Yes	51,938	(37.07)	1388	(41.85)	
Recruitment period					< 0.0001
2012–2015	45,307	(32.34)	1475	(44.47)	
2016–2019	57,543	(41.07)	1185	(35.73)	
2020–2022	37,258	(26.59)	657	(19.81)	

Abbreviations: HCV, Hepatitis C Virus; BMI, Body Mass Index; NTD, National Taiwan Dollar

^a^: Percentages among the none HBV and/or HCV infected participants

^b^: Percentages among the HCV infected participants

^c^: Comparing the Non-viral and HCV infected groups

### Association between hepatic viral infections and cancer

Univariate analysis revealed that HBV was associated with both liver cancer (OR = 8.39, 95% CI = 4.15–16.98, *P* < 0.001) and ovarian cancer (OR = 4.15, 95% CI = 1.81–9.47, *P* = 0.001). Interestingly, after adjusting for age group, sex, education level, marital status, maternal and paternal origin, residence, BMI, income level, family history of cancer, alcohol consumption, smoking experience, betel nut experience, hyperlipidemia, and diabetes, the associations between HBV infection and liver (adjusted odds ratio (aOR) = 6.60, 95% CI = 3.21–13.57, *P* < 0.001) and ovarian (aOR = 4.63, 95% CI = 1.98–10.83, *P* = 0.001) cancer remained statistically significant (Table 3).

**Table 3:** The association between HBV infection and cancer

Cancer sites	No HBV		HBV- infected		OR	95% CI	*P*	aOR^*^	95% CI	*P*
	N	‱	N	‱						
Liver	**16**	**1.14**	**15**	**9.57**	**8.39**	**4.15–16.98**	** < 0.001**	**6.60**	**3.21–13.57**	** < 0.001**
Lung	46	3.28	2	1.28	0.39	0.09–1.60	0.191	0.36	0.09–1.47	0.153
Breast^a^	257	28.58	31	33.91	1.19	0.82–1.72	0.368	1.08	0.74–1.58	0.691
Gastric	9	0.64	1	0.64	0.99	0.13–7.84	0.995	0.70	0.09–5.60	0.737
Colorectal	64	4.57	8	5.11	1.12	0.54–2.33	0.766	1.01	0.48–2.13	0.972
Nasopharyngeal	16	1.14	2	1.28	1.12	0.26–4.86	0.882	1.11	0.25–4.97	0.894
Prostate^b^	22	4.38	1	1.53	0.35	0.05–2.59	0.304	0.30	0.04–2.25	0.241
Cervical^a^	141	15.68	20	21.87	1.40	0.87–2.23	0.163	1.26	0.78–2.02	0.808
Ovarian^a^	**19**	**2.11**	**8**	**8.75**	**4.15**	**1.81–9.47**	**0.001**	**4.63**	**1.98–10.83**	**0.001**
Uterine^a^	48	5.34	8	8.75	1.64	0.78–3.47	0.196	1.53	0.72–3.26	0.274
Oral	22	1.57	1	0.64	0.41	0.06–3.02	0.379	0.38	0.05–2.83	0.342
Bone	10	0.71	2	1.28	1.79	0.39–8.16	0.453	1.56	0.34–7.16	0.876
Non-Hodgkin’s	33	2.36	3	1.91	0.81	0.25–2.65	0.732	0.68	0.21–2.23	0.525
Thyroid	62	4.43	10	6.38	1.44	0.74–2.81	0.282	1.33	0.68–2.60	0.410
Skin	12	0.86	1	0.64	0.75	0.10–5.73	0.778	0.72	0.09–5.64	0.757
Kidney	6	0.43	2	1.28	2.98	0.60–14.77	0.181	2.68	0.54–13.33	0.229
Brain	8	0.57	2	1.28	2.24	0.48–10.53	0.309	2.04	0.42–10.01	0.381
Bladder	13	0.93	0	0.00	–	–	–	–	–	–
Other^c^	42	3.00	1	0.64	0.21	0.03–1.55	0.126	0.63	0.19–2.05	0.444

Bold results indicate statistically significant findings with a *P* value less than 0.05

Abbreviations: HBV, Hepatitis B Virus; OR, Odds Ratio; CI, Confidence Interval, aOR; Adjusted Odds ratio

* Adjusted for age group, sex, education level, marital status, maternal and paternal origin, residence, BMI, income level, family history of cancer, alcohol drinking, smoking experience, betel nut experience, recruitment period, hyperlipidemia, and diabetes

^a^: Females only

^b^: Males only

^c^: Other; throat cancer, retroperitoneal cancer, testicular cancer, neuroblastoma, cholangiocarcinoma, thymus cancer, spleen cancer, gall bladder cancer, ureteral cancer, vaginal cancer, esophageal cancer, bone marrow cancer and cancer of the head and neck

Analysis of HCV infection revealed significant associations with liver cancer (OR = 7.93, 95% CI = 2.31–27.22, *P* = 0.001), ovarian cancer (OR = 4.53, 95% CI = 1.06–19.46, *P* = 0.042), and kidney cancer (OR = 14.09, 95% CI = 2.84–69.83, *P* = 0.001) during the univariate analysis. Upon incorporation of necessary adjustments for significant covariates, HCV infection was still observed to be associated with liver cancer (aOR = 4.90, 95% CI = 1.37–17.53, *P* = 0.015), ovarian cancer (aOR = 8.50, 95% CI = 1.78–40.69, *P* = 0.007), and kidney cancer (aOR = 12.89, 95% CI = 2.41–69.01, *P* = 0.003). (Table 4).

**Table 4:** The association between HCV infection and cancer

Cancer sites	No HCV		HCV- infected		OR	95% CI	*P*	aOR^*^	95% CI	*P*
	N	‱	N	‱						
Liver	**16**	**1.14**	**3**	**9.04**	**7.93**	**2.31–27.22**	**0.001**	**4.90**	**1.37–17.53**	**0.015**
Lung	46	3.28	2	6.03	1.84	0.45–7.57	0.400	1.59	0.38–6.68	0.530
Breast^a^	257	28.58	5	23.91	0.84	0.36–2.03	0.693	0.76	0.31–1.88	0.552
Gastric	9	0.64	0	0.00	–	–	–	–	–	–
Colorectal	64	4.57	1	3.01	0.66	0.09–4.76	0.680	0.44	0.06–3.24	0.423
Nasopharyngeal	16	1.14	0	0.00	–	–	–	–	–	–
Prostate^b^	22	4.38	1	8.17	1.86	0.25–13.82	0.543	1.23	0.16–9.43	0.841
Cervical^a^	141	15.68	4	21.87	1.22	0.45–3.30	0.694	1.13	0.41–3.13	0.817
Ovarian^a^	**19**	**2.11**	**2**	**9.56**	**4.53**	**1.06–19.46**	**0.042**	**8.50**	**1.78–40.69**	**0.007**
Uterine^a^	48	5.34	0	0.00	–	–	–	–	–	–
Oral	22	1.57	0	0.00	–	–	–	–	–	–
Bone	10	0.71	1	3.01	4.23	0.54–33.01	0.170	2.98	0.57–24.20	0.307
Non-Hodgkin’s	33	2.36	0	0.00	–	–	–	–	–	–
Thyroid	62	4.43	1	3.01	0.68	0.10–4.91	0.705	0.65	0.09–4.75	0.672
Skin	12	0.86	1	3.01	3.52	0.46–27.09	0.226	4.71	0.58–38.43	0.148
Kidney	**6**	**0.43**	**2**	**6.03**	**14.09**	**2.84–69.83**	**0.001**	**12.89**	**2.41–69.01**	**0.003**
Brain	8	0.57	0	0.00	–	–	–	–	–	–
Bladder	13	0.93	0	0.00	–	–	–	–	–	–
Other^c^	42	3.00	0	0.00	–	–	–	–	–	–

Bold results indicate statistically significant findings with a *P* value less than 0.05

Abbreviations: HCV, Hepatitis C Virus; OR, Odds Ratio; CI, Confidence Interval, aOR; Adjusted Odds ratio

*: Adjusted for age group, education level, marital status, maternal and paternal origin, residence, BMI, income level, family history of cancer, alcohol drinking, smoking experience, betel nut experience, exercise regularity, recruitment period, hypertension, and diabetes

^a^: Females only

^b^: Males only

^c^^:^ Other; throat cancer, retroperitoneal cancer, testicular cancer, neuroblastoma, cholangiocarcinoma, thymus cancer, spleen cancer, gall bladder cancer, ureteral cancer, vaginal cancer, esophageal cancer, bone marrow cancer and cancer of the head and neck

### Sensitivity analysis

Table 5 shows the association between HBV and cancer after the omission of the dual infection group. In both the univariate and multivariate analyses, significant associations remained for liver cancer (aOR = 6.85, 95% CI = 3.33–14.10, *P* < 0.001) and ovarian cancer (aOR = 4.76, 95% CI = 2.04–11.12, *P* = 0.001).

**Table 5:** The association between HBV ‘alone’ and cancer

Cancers	No HBV		HBV only		OR	95% CI	*P*	aOR^*^	95% CI	*P*
	N	‱	N	‱						
Liver	**16**	**1.14**	**15**	**9.80**	**8.59**	**4.25–17.38**	** < 0.001**	**6.85**	**3.33–14.10**	** < 0.001**
Lung	46	3.28	2	1.31	0.40	0.10–1.64	0.202	0.37	0.09–1.52	0.166
Breast^a^	257	28.58	30	33.61	1.18	0.81–1.72	0.400	1.08	0.73–1.59	0.705
Gastric	9	0.64	1	0.65	1.02	0.13–8.03	0.987	0.72	0.09–5.72	0.752
Colorectal	64	4.58	8	5.23	1.14	0.55–2.39	0.719	1.07	0.51–2.26	0.853
Nasopharyngeal	16	1.14	2	1.31	1.14	0.26–4.98	0.857	1.13	0.25–5.09	0.869
Prostate^b^	22	4.38	1	1.57	0.36	0.05–2.65	0.315	0.31	0.04–2.35	0.259
Cervical^a^	141	15.68	18	20.16	1.29	0.79–2.10	0.314	1.16	0.70–1.91	0.561
Ovarian^a^	**19**	**2.11**	**8**	**8.96**	**4.25**	**1.89–9.70**	**0.001**	**4.76**	**2.04–11.12**	**0.001**
Uterine and corpus^a^	48	5.34	8	8.96	1.68	0.79–3.55	0.175	1.60	0.75–3.42	0.224
Oral	22	1.57	1	0.65	0.42	0.06–3.09	0.391	0.39	0.05–2.91	0.357
Bone	10	0.71	1	0.65	0.92	0.12–7.15	0.933	0.83	0.11–6.52	0.861
Non-Hodgkin’s	33	2.36	3	1.96	0.83	0.26–2.71	0.761	0.70	0.22–2.29	0.559
Thyroid	62	4.43	10	6.54	1.48	0.76–2.88	0.253	1.37	0.70–2.69	0.358
Skin	12	0.86	1	0.65	0.76	0.10–5.87	0.795	0.76	0.10–5.88	0.788
Kidney	6	0.43	2	1.31	3.05	0.62–15.12	0.172	2.78	0.56–13.88	0.212
Brain	8	0.57	2	1.31	2.29	0.48–10.78	0.295	2.11	0.43–10.38	0.358
Bladder	13	1.21	0	–	–	–	–	–	–	–
Other^c^	42	3.00	1	0.65	0.22	0.03–1.58	0.132	0.44	0.11–1.82	0.255

Bold results indicate statistically significant findings with a *P* value less than 0.05

Abbreviations: HBV, Hepatitis B Virus; OR, Odds Ratio; aOR, Adjusted Odds Ratio; CI, Confidence Interval

*: Adjusted for age group, sex, education level, marital status, maternal and paternal origin, residence, BMI, income level, family history of cancer, alcohol drinking, smoking experience, betel nut experience, exercise regularity, recruitment period, hyperlipidemia, and diabetes

^a^: Females only

^b^: Males only

^c^: Other; throat cancer, retroperitoneal cancer, testicular cancer, neuroblastoma, cholangiocarcinoma, thymus cancer, spleen cancer, gall bladder cancer, ureteral cancer, vaginal cancer, esophageal cancer, bone marrow cancer and cancer of the head and neck

Similarly, for HCV infection, both the univariate and multivariate analyses showed that the association remained for liver cancer (aOR = 5.94, 95% CI = 1.66–21.28, *P* = 0.006), ovarian cancer (aOR = 9.99, 95% CI = 2.08–47.94, *P* = 0.004), and kidney cancer (aOR = 15.39, 95% CI = 2.82–84.03, *P* = 0.002) (Table 6).

**Table 6:** The association between HCV ‘alone’ and cancer

Cancers	No HCV		HCV only		OR	95% CI	*P*	aOR^*^	95% CI	*P*
	N	‱	N	‱						
Liver	**16**	**1.14**	**3**	**10.15**	**8.90**	**2.59–30.55**	**0.001**	**5.94**	**1.66–21.28**	**0.006**
Lung	46	3.28	2	6.77	2.06	0.50–8.50	0.316	1.85	0.44–7.82	0.403
Breast^a^	257	28.58	4	21.33	0.75	0.28–2.01	0.562	0.69	0.25–1.90	0.475
Gastric	9	0.64	0	0.00	–	–	–	–	–	–
Colorectal	64	4.57	1	3.38	0.74	0.10–5.34	0.766	0.54	0.07–3.95	0.544
Nasopharyngeal	16	1.14	0	0.00	–	–	–	–	–	–
Prostate^b^	22	4.38	1	9.26	2.12	0.29–15.69	0.464	1.48	0.19–11.36	0.708
Cervical^a^	141	15.68	2	10.67	0.68	0.17–2.75	0.590	0.63	0.15–2.61	0.526
Ovarian^a^	**19**	**2.11**	**2**	**10.67**	**5.05**	**1.18–21.71**	**0.029**	**9.99**	**2.08–47.94**	**0.004**
Uterine^a^	48	3.43	0	0.00	–	–	–	–	–	–
Oral	22	1.57	0	0.00	–	–	–	–	–	–
Bone	10	0.71	0	0.00	–	–	–	–	–	–
Non-Hodgkin’s	33	2.36	0	0.00	–	–	–	–	–	–
Thyroid	62	4.43	1	3.38	0.77	0.11–5.52	0.791	0.79	0.11–5.75	0.812
Skin	12	0.86	1	3.38	3.95	0.51–30.40	0.187	6.08	0.74–49.85	0.093
Kidney	**6**	**0.43**	**2**	**6.77**	**15.82**	**3.19–78.39**	**0.001**	**15.39**	**2.82–84.03**	**0.002**
Brain	8	0.57	0	0.00	**–**	**–**	**–**	**–**	**–**	**–**
Bladder	13	0.93	0	0.00	**–**	**–**	**–**	**–**	**–**	**–**
Other^c^	42	3.00	0	0.00	**–**	**–**	**–**	**–**	**–**	**–**

Bold results indicate statistically significant findings with a *P* value less than 0.05

Abbreviations: HCV, Hepatitis C Virus; OR, Odds Ratio; aOR, Adjusted Odds Ratio; CI, Confidence Interval

^*^: Adjusted for age group, sex, education level, marital status, maternal and paternal origin, residence, BMI, income level, family history of cancer, alcohol drinking, smoking experience, betel nut experience, exercise regularity, recruitment period, hyperlipidemia, and diabetes

^a^: Females only

^b^: Males only

^c^: Other; throat cancer, retroperitoneal cancer, testicular cancer, neuroblastoma, cholangiocarcinoma, thymus cancer, spleen cancer, gall bladder cancer, ureteral cancer, vaginal cancer, esophageal cancer, bone marrow cancer and cancer of the head and neck

## Discussion

In the current study, we observed that hepatitis viral infections were associated not only with liver cancer but also with some extrahepatic cancer types. In particular, the extrahepatic cancers observed to be associated with HCV infection were ovarian and kidney cancers, whereas those with HBV infection were ovarian cancer. In the sensitivity analysis, the same associations were observed between HBV and HCV infections and cancer. This finding reinforces the robustness of our findings.

A few recent studies have examined the association between HBV infection and intra- and extrahepatic cancer. In these studies, the association with liver cancer was consistent, but the associated extrahepatic cancers varied among different studies. A study by Kamiza et al. revealed associations between HBV infection and an increased likelihood of hepatocellular carcinoma and ovarian cancer [12], similar to our study. He additionally reported associations between colorectal cancer, gallbladder and extrahepatic bile duct cancer, pancreatic cancer, renal cancer, and non-Hodgkin’s lymphoma using the Taiwan National Health Insurance Research Database (NHIRD) in a retrospective cohort study [12]. Similar findings were observed by Spradling et al. who demonstrated a higher incidence of ovarian and hepatocellular cancers among HBV-infected participants in a cohort study in the United States. Other incident extrahepatic cancers included gastric, cholangiocarcinoma, neuroendocrine, and non-Hodgkin cancers [18]. A Chinese-based longitudinal study revealed associations between HBV infection and the risk of liver cancer, stomach cancer, colorectal cancer, oral cancer, pancreatic cancer, and lymphoma in a prospective cohort study using a nested case‒control study [13]. The extrahepatic associations revealed in this study are in contrast to our findings. Another study on the elderly population in the United States reported significant associations between HBV infection and an increased risk of stomach cancer, cancer of the anus, liver cancer, intrahepatic bile duct cancer, nasopharynx cancer, and myelodysplastic syndrome [14]. All the above studies showed consistent associations with liver cancer but different extrahepatic cancer types.

Several studies have examined the relationship between HCV infection and various types of cancer. Our findings are consistent with those of Kamiza et al. who observed significant associations between liver and ovarian cancer. The author also revealed associations with other extrahepatic cancers, such as gallbladder and extrahepatic bile duct cancers, and non-Hodgkin’s lymphoma [12]. Another study revealed significant associations between HCV infection and the risk of renal cancer in a hospital-based cohort study conducted in southeastern Michigan [19], consistent with our study. Similarly, a Taiwanese study based on the NHIRD revealed an association between kidney cancer and HCV infection [20]. A large study of the Canadian population revealed an elevated risk of some extrahepatic cancers associated with HCV infection, including liver, anal, esophageal, larynx, lung, and oral cancer [21]. Similarly, the associations with HCV examined in the above studies revealed significant associations with liver cancer and various extrahepatic cancers.

The coherent findings with the Taiwanese-based studies could be attributed to the fact that the studies were carried out in the same population group with similar exposures, lifestyle behaviors, diet, and genetic patterns. Otherwise, both HBV and HCV are known to be risk factors for liver cancer, and this association is expected. The inconsistencies observed could be attributed to differences in study designs, database types, populations, control of different confounders, and different analytical strategies. All the above studies used longitudinal study designs, unlike the current study. The different populations explored have different risk factors due to differences in diet patterns, lifestyle behaviors, environmental exposures, and comorbidities, making it imperative to observe different extrahepatic manifestations.

HBV and HCV are oncogenic viruses, particularly in liver cancer [22]. The oncogenic role of these viruses in hepatocytes is believed to be both direct and indirect. The direct mechanism is by forming covalently closed circular deoxyribonucleic acid (cccDNA) structures in hepatocytes, leading to chronic injury and inflammation [23, 24], and indirectly by altering cellular signal transduction pathways [23, 25, 26]. However, few studies have explored the mechanism of carcinogenesis of HBV and HCV in extracellular cancers. Song et al. reported the presence of HBV DNA and hepatitis B X protein in stomach and pancreatic cancer tissues but not in lung cancer tissues [13]. The presence of HCV ribonucleic acid (RNA) has also been detected in ovarian cancer tissue [27, 28]. Nucleic acids and core proteins of HCV have also been isolated from kidney tissue [29, 30]. HCV RNA was isolated in 65% of the glomerular tissue analyzed, and HCV core protein was identified in 77.5% of the tubules and glomeruli [29]. The presence of these biomarkers in extrahepatic cancer tissues could indicate the likelihood of viral replication in these cells, leading to chronic inflammation and cancer development.

This study provides evidence of an association between hepatic viral infections (HBV and HCV) and both hepatic and extrahepatic cancer. However, it had several strengths and limitations. The first strength of this study was the use of a large database covering a wide geographic location of the Taiwanese population. Second, the study used a highly sensitive method for the diagnosis of HCV and HBV cases, ECLIA, which has a sensitivity greater than 99% and a specificity of 100% [31]. This implies a high diagnostic accuracy for HBV and HCV infections. In addition, the study controlled for a wide range of confounding variables, including sociodemographics, lifestyle behaviors, and comorbidities, providing an opportunity to control a wide range of risk factors.

Our study had some limitations. Being cross-sectional in nature, we were unable to infer causality due to the limitations of the study design. The study only presents the likely association between hepatic viral infections and cancers and an opportunity to explore them further. Second, the generalizability of the study may be limited because of the small number of cancer events in the baseline study. However, this is validated by the fact that associations with liver cancer were observed to be significant (HBV and HCV are known risk factors for hepatocellular cancer) [32–34]. The findings of the study were also observed to be consistent with previous research [12, 19, 20]. Third, the influence of likely confounding from other viral infections such as human immunodeficiency virus [35] and human papilloma virus [36, 37] could not be ruled out. Fourth, the extended coverage time of the cross-sectional study, spanning from 2012 to 2022 introduces the possibility of changes in various confounding factors over time, which could influence the observed associations. While we accounted for the prolonged recruitment span in our analysis, it is crucial to exercise caution in generalizing the findings to different periods. Further research with a more focused temporal scope is recommended to validate and extend our observations. Finally, the cases of cancer in the TWB database were based on a survey, which may not be the most accurate way to diagnose cancer. Future studies should consider linking this database to other databases, such as the cancer registry or the NHIRD, to ensure a more comprehensive diagnosis of cancer. However, these cancer results can be relied upon because patients are less likely to lie about their cancer status [38].

In conclusion, hepatic viral infections (HBV and HCV) were not only associated with an increased likelihood of hepatocellular cancer but also with extrahepatic cancers such as ovarian and kidney cancers. The TWB database provides a great opportunity to investigate these associations further in a longitudinal study due to the large sample size, long follow-up period, and potential for controlling a wide range of likely confounding variables.

## Data Availability

The data that support the findings of this study are not openly available due to licenses\restrictions and can be available from the Taiwan Biobank upon submission of a formal proposal. Requests to access these datasets should be directed to biobank@gate.sinica.edu.tw.

## Abbreviations

aOR: Adjusted odds ratio
BMI: Body mass index
CI: Confidence interval
DNA: Deoxyribonucleic acid
ECLIA: Electrochemiluminescence assay
HBsAg: Hepatitis B surface antigen
HBV: Hepatitis B virus
HCV: Hepatitis C virus
NHIRD: National health insurance research database
NTD: National Taiwan dollar
OR: Odds ratio
RNA: Ribonucleic acid
TWB: Taiwan biobank
WHO: World Health Organization

## References

[1] World Health Organization: Hepatitis. 2023. https://www.who.int/health-topics/hepatitis#tab=tab_1. Accessed 18 March 2023.

[2] World Health Organization: Hepatitis B. 2022. https://www.who.int/news-room/fact-sheets/detail/hepatitis-b. Accessed 26 May 2023.

[3] World Health Organization: Hepatitis C. 2022. https://www.who.int/news-room/fact-sheets/detail/hepatitis-c. Accessed 26 May 2023.

[4] Hu YC, Yeh CC, Chen RY, Su CT, Wang WC, Bai CH, et al. Seroprevalence of hepatitis B virus in Taiwan 30 years after the commencement of the national vaccination program. PeerJ. 2018. 10.7717/peerj.4297. 29472994 10.7717/peerj.4297 PMC5817935

[5] Wait S, Chen DS. Toward the eradication of hepatitis B in Taiwan. Kaohsiung J Med Sci. 2012. 10.1016/j.kjms.2011.10.027. 22226055 10.1016/j.kjms.2011.10.027 PMC11916147

[6] Chen CH, Yang PM, Huang GT, Lee HS, Sung JL, Sheu JC. Estimation of seroprevalence of hepatitis B virus and hepatitis C virus in Taiwan from a large-scale survey of free hepatitis screening participants. J Formos Med Assoc. 2007. 10.1016/S0929-6646(09)60231-X. 17339159 10.1016/S0929-6646(09)60231-X

[7] Liu CL, Chang HF, Huang JJ, Chou YM. Prevention and control of hepatitis B in Taiwan. Epidemiol Bull. 2016. 10.6525/TEB.20160719.32(14).001.

[8] Yu ML, Chen PJ, Dai CY, Hu TH, Huang CF, Huang YH, et al. Taiwan consensus statement on the management of hepatitis C: part (I) general population. J Formos Med Assoc. 2020. 10.1016/j.jfma.2020.04.003. 32359879 10.1016/j.jfma.2020.04.003

[9] Yang JF, Lin CI, Huang JF, Dai CY, Lin WY, Ho CK, et al. Viral hepatitis infections in southern Taiwan: a multicenter community-based study. Kaohsiung J Med Sci. 2010. 10.1016/S1607-551X(10)70073-5. 20837342 10.1016/S1607-551X(10)70073-5 PMC11916219

[10] Yu ML, Yeh ML, Tsai PC, Huang CI, Huang JF, Huang CF, et al. Huge gap between clinical efficacy and community effectiveness in the treatment of chronic hepatitis C: a nationwide survey in Taiwan. Medicine (Baltimore). 2015. 10.1097/MD.0000000000000690. 25837762 10.1097/MD.0000000000000690 PMC4554019

[11] Su FH, Le TN, Muo CH, Te SA, Sung FC, Yeh CC. Chronic hepatitis B virus infection associated with increased colorectal cancer risk in Taiwanese population. Viruses. 2020. 10.3390/v12010097. 31947702 10.3390/v12010097 PMC7019239

[12] Kamiza AB, Su FH, Wang WC, Sung FC, Chang SN, Yeh CC. Chronic hepatitis infection is associated with extrahepatic cancer development: a nationwide population-based study in Taiwan. BMC Cancer. 2016. 10.1186/s12885-016-2918-5. 27821099 10.1186/s12885-016-2918-5 PMC5100218

[13] Song C, Lv J, Liu Y, Chen JG, Ge Z, Zhu J, et al. Associations between hepatitis B virus infection and risk of all cancer types. JAMA Netw Open. 2019. 10.1001/jamanetworkopen.2019.5718. 31199446 10.1001/jamanetworkopen.2019.5718 PMC6575146

[14] Mahale P, Torres HA, Kramer JR, Hwang LY, Li R, Brown EL, Engels EA. Hepatitis C virus infection and the risk of cancer among elderly US adults: a registry-based study. Cancer. 2017. 10.1002/cncr.30559. 28117886 10.1002/cncr.30559 PMC6295146

[15] The Overseas Community Affairs Council (R.O.C) Taiwan. Cancer remains leading cause of death in Taiwan for 40th year. 2022. https://www.ocac.gov.tw/OCAC/Eng/Pages/Detail.aspx?nodeid=329&pid=41847299. Accessed 26 May 2023.

[16] Feng YA, Chen CY, Chen TT, Kuo PH, Hsu YH, Yang HI, et al. Taiwan biobank: a rich biomedical research database of the Taiwanese population. Cell Genom. 2022. 10.1016/j.xgen.2022.100197. 36776991 10.1016/j.xgen.2022.100197 PMC9903657

[17] Chang LT, Liu IJ, Chang TY, Hong GB, Lin LY, Chuang HC, et al. Association of long-term indoor exposure to fine particles with pulmonary effects in Northern Taiwan. Sci Total Environ. 2022. 10.1016/j.scitotenv.2022.153097. 35041956 10.1016/j.scitotenv.2022.153097

[18] Spradling PR, Xing J, Zhong Y, Rupp LB, Moorman AC, Lu M, et al. Incidence of malignancies among patients with chronic hepatitis B in US health care organizations, 2006–2018. J Infect Dis. 2022. 10.1093/infdis/jiac011. 35039863 10.1093/infdis/jiac011

[19] Gordon SC, Moonka D, Brown KA, Rogers C, Huang MAY, Bhatt N, et al. Risk for renal cell carcinoma in chronic hepatitis C infection. Cancer Epidemiol Biomarkers Prev. 2010. 10.1158/1055-9965.EPI-09-1275. 20332260 10.1158/1055-9965.EPI-09-1275

[20] Lin YS, Yeh CC, Lin YC, Wei X. Kidney Cancer linked to chronic hepatitis in the Asia-Pacific: a population-based analysis. Am J Nephrol. 2017. 10.1159/000453045. 27866208 10.1159/000453045

[21] Darvishian M, Tang T, Wong S, Binka M, Yu A, Alvarez M, et al. Chronic hepatitis C infection is associated with higher incidence of extrahepatic cancers in a Canadian population based cohort. Front Oncol. 2022. 10.3389/fonc.2022.983238. 36313680 10.3389/fonc.2022.983238 PMC9609415

[22] Tempera I, Lieberman PM. Oncogenic viruses as entropic drivers of cancer evolution. Front Virol. 2021. 10.3389/fviro.2021.753366. 35141704 10.3389/fviro.2021.753366 PMC8822580

[23] Bouchard MJ, Navas-Martin S. Hepatitis B and C virus hepatocarcinogenesis: lessons learned and future challenges. Cancer Lett. 2011. 10.1016/j.canlet.2010.11.014. 21168955 10.1016/j.canlet.2010.11.014 PMC3071446

[24] El-Serag HB, Rudolph KL. Hepatocellular carcinoma: epidemiology and molecular carcinogenesis. Gastroenterology. 2007. 10.1053/j.gastro.2007.04.061. 17570226 10.1053/j.gastro.2007.04.061

[25] Bartosch B, Thimme R, Blum HE, Zoulim F. Hepatitis C virus-induced hepatocarcinogenesis. J Hepatol. 2009. 10.1016/j.jhep.2009.05.008. 19545926 10.1016/j.jhep.2009.05.008

[26] Roberts LR, Gores GJ. Hepatocellular carcinoma: molecular pathways and new therapeutic targets. Semin Liver Dis. 2005. 10.1055/s-2005-871200. 15918149 10.1055/s-2005-871200

[27] Böcher WO, Löhr HF, Steegmüller KW, Störkel S, Jäger U, Büschenfelde KHMZ, et al. Detection of hepatitis C virus replication in ovarian metastases of a patient with hepatocellular carcinoma. J Hepatol. 1994. 10.1016/s0168-8278(94)80135-5. 7963421 10.1016/s0168-8278(94)80135-5

[28] Xu F, Zhu X, Han T, You X, Liu F, Ye L, et al. The oncoprotein hepatitis B X-interacting protein promotes the migration of ovarian cancer cells through the upregulation of S-phase kinase-associated protein 2 by Sp1. Int J Oncol. 2014. 10.3892/ijo.2014.2411. 24788380 10.3892/ijo.2014.2411

[29] Sansonno D, Lauletta G, Montrone M, Schena FP, Dammacco F. Hepatitis C virus RNA and core protein in kidney glomerular and tubular structures isolated with laser capture microdissection. Clin Exp Immunol. 2005. 10.1111/j.1365-2249.2005.02778.x. 15932511 10.1111/j.1365-2249.2005.02778.x PMC1809381

[30] Ko HM, Hernandez-Prera JC, Zhu H, Dikman SH, Sidhu HK, Ward SC, et al. Morphologic features of extrahepatic manifestations of hepatitis C virus infection. Clin Dev Immunol. 2012. 10.1155/2012/740138. 22919404 10.1155/2012/740138 PMC3420144

[31] Wadood M, Usman M. Comparative analysis of electrochemiluminescence assay and chemiluminescent microparticle immunoassay for the screening of hepatitis C. Indian J Hematol Blood Transfus. 2019. 10.1007/s12288-018-0968-3. 30828160 10.1007/s12288-018-0968-3 PMC6369078

[32] Ringelhan M, McKeating JA, Protzer U. Viral hepatitis and liver cancer. Philos Trans R Soc Lond B Biol Sci. 2017. 10.1098/rstb.2016.0274. 28893941 10.1098/rstb.2016.0274 PMC5597741

[33] Fattovich G. Progression of hepatitis B and C to hepatocellular carcinoma in Western countries. Hepatogastroenterology. 1998;45(Suppl 3):1206–13. 9730376

[34] Amin J, Dore GJ, O’Connell DL, Bartlett M, Tracey E, Kaldor JM, et al. Cancer incidence in people with hepatitis B or C infection: a large community-based linkage study. J Hepatol. 2006. 10.1016/j.jhep.2006.02.014. 16684579 10.1016/j.jhep.2006.02.014

[35] Clifford GM, Rickenbach M, Polesel J, Maso LD, Steffen I, Ledergerber B, et al. Influence of HIV-related immunodeficiency on the risk of hepatocellular carcinoma. AIDS. 2008. 10.1097/QAD.0b013e32831103ad. 18832877 10.1097/QAD.0b013e32831103ad

[36] Ferber MJ, Montoya DP, Yu C, Aderca I, McGee A, Thorland EC, et al. Integrations of the hepatitis B virus (HBV) and human papillomavirus (HPV) into the human telomerase reverse transcriptase (hTERT) gene in liver and cervical cancers. Oncogene. 2003. 10.1038/sj.onc.1206528. 12802289 10.1038/sj.onc.1206528

[37] Kao SS, Li CJ, Wei JC, Lin CL, Chang R, Hung YM. Human papillomavirus infection is associated with decreased risk of hepatocellular carcinoma in chronic hepatitis C patients: Taiwan nationwide matched cohort study. Cancers (Basel). 2022. 10.3390/cancers14051289. 35267595 10.3390/cancers14051289 PMC8909203

[38] Jiang Y, Liu C, Li JY, Huang MJ, Yao WX, Zhang R, et al. Different attitudes of Chinese patients and their families toward truth telling of different stages of cancer. Psychooncology. 2007. 10.1002/pon.1156. 17285684 10.1002/pon.1156

